# Dipeptidyl-Peptidase 4 Inhibitor Sitagliptin Ameliorates Hepatic Insulin Resistance by Modulating Inflammation and Autophagy in ob/ob Mice

**DOI:** 10.1155/2018/8309723

**Published:** 2018-07-19

**Authors:** Wenbin Zheng, Jing Zhou, Shasha Song, Wen Kong, Wenfang Xia, Lulu Chen, Tianshu Zeng

**Affiliations:** ^1^Department of Endocrinology, Union Hospital, Tongji Medical College, Huazhong University of Science and Technology, Wuhan 430022, China; ^2^Department of Nutrition and Food Science, Texas A&M University, College Station, TX, USA

## Abstract

Obesity and type 2 diabetes are the most common metabolic diseases globally. They are associated with inflammation, oxidative stress, autophagy, and insulin resistance. Sitagliptin, a dipeptidyl-peptidase 4 inhibitor, has been reported to show multiple biological activities beyond the antidiabetic property. This study was aimed at investigating the effect of sitagliptin on hepatic steatosis, insulin resistance, inflammation, and autophagy and exploring the underlying molecular mechanism. In the current study, ob/ob mice, a mouse model of genetic obesity and diabetes, were administered via gavage with sitagliptin 50 mg/kg daily for 4 weeks. Changes in glycolipid metabolism, inflammatory responses, and autophagy in the liver were evaluated. Body weight gain, lipid metabolic disorder, and hepatic steatosis as well as systemic and hepatic insulin sensitivity in ob/ob mice were significantly attenuated after sitagliptin treatment. Furthermore, sitagliptin decreased inflammatory responses by regulating macrophage M1/M2 polarization and inhibiting the activities of NF-*κ*B and JNK. Moreover, sitagliptin increased the levels of phosphorylation of AMPK and decreased those of mTOR. This study indicates that sitagliptin significantly ameliorates the development of hepatic steatosis and insulin resistance in ob/ob mice by inhibiting inflammatory responses and activating autophagy via AMPK/mTOR signaling pathway.

## 1. Introduction

The prevalence of diabetes and obesity has increased tremendously worldwide, mainly because of increased energy intake and decreased energy expenditure. Chronic, low-grade, metabolic inflammation is a key component in the pathogenesis of obesity and associated disorders such as insulin resistance and type 2 diabetes (T2D) [1]. Macrophage accumulation increases in adipose tissue as obesity develops and appears to shift towards proinflammatory M1 macrophages [2]. It has been reported that compared with lean controls, adipose tissues of the obese individuals secrete elevated levels of inflammatory cytokines such as tumor necrosis factor- (TNF-) *α* and interleukin- (IL-) 6, which impair insulin signaling and cause insulin resistance [3–5]. In addition, genetic ablation of Jun N-terminal kinase (JNK) or I*κ*B kinase (IKK*β*) protected mice from high-fat diet- (HFD-) induced inflammation and insulin resistance [6, 7], demonstrating that these inflammatory signaling pathways in macrophages are crucial regulators in metabolic dysfunction.

The liver plays a critical role in the regulation of metabolic homeostasis, controlling not only gluconeogenesis and glycogen storage but also great amounts of lipogenesis and cholesterol synthesis and secretion. Although extensive studies focused on adipose tissue macrophages in the context of insulin resistance, liver macrophages have been shown as a contributor to the production of inflammatory mediators that promote insulin resistance in hepatocytes. Several studies have proposed that during obesity, the hepatic inflammation was induced, associated with a significant increase in liver macrophages [8, 9]. Chemical ablation of hepatic macrophages protects mice from HFD-induced insulin resistance and hepatic steatosis, revealing the importance of these cells in the development of metabolic disorders [9, 10]. Thus, inflammatory activation of liver macrophages contributes importantly to the pathogenesis of obesity-induced insulin resistance and hepatic steatosis.

Autophagy is a cellular degradation pathway maintaining cellular homeostasis. Consistent evidence has revealed the relationship between inflammation and the onset and development of metabolic diseases, and recent developments demonstrate a pivotal role for the autophagy pathway under inflammatory conditions. Therefore, in recent years, research on autophagy has begun to focus on its interaction with metabolic inflammation, but the precise relationship and mechanism are poorly understood [11, 12]. Several studies have shown that high-fat diet for 16 weeks resulted in marked reduction of LC3, Beclin 1, and Atg7 protein levels in the liver [13]. Insulin signaling was attenuated in the liver and adipose tissues of macrophage-specific Atg7KO mice, accompanied with improvement of hepatic steatosis [14]. A recent research, in which macrophage-specific Atg5KO mice increased proinflammatory M1 macrophage polarization under HFD conditions in combination with LPS treatment, has supported the role of autophagy in regulating the macrophage activation [15]. Our previous experiments also showed that blocking the hepatic inflammatory pathway led to the macrophage polarization imbalance, accompanied with an increase in liver autophagy [16].

Dipeptidyl-peptidase 4 (DPP-4), ubiquitously expressed on the membranes of many cell types, can serve as a mediator of inflammation and metabolism [17, 18]. DPP-4 inhibitors prevent the degradation of glucagon-like peptide 1 (GLP-1) and glucose-dependent insulinotropic peptide (GIP) and play important roles in both postprandial and long-term glucose homeostasis [19]. Numerous studies have demonstrated that DPP-4 inhibitors improve insulin sensitivity, and there is increasing evidence that the DPP-4 inhibitors have anti-inflammatory effect. One study showed that treatment with sitagliptin reduced plasma levels of proinflammatory markers in type 2 diabetes patients [20]. Zhuge et al. suggested that linagliptin attenuated obesity-related insulin resistance and inflammation by regulating both macrophage recruitment and M1/M2 status within white adipose tissue (WAT) and the liver in diet-induced obese (DIO) mice [21]. Another important study showed that both DPP4 inhibition by orally administered sitagliptin and hepatocyte DPP4 silencing for 4 weeks lowered blood glucose and improved oral glucose tolerance, but only hepatocyte-specific deletion of DPP4 increased insulin-induced p-AKT in VAT and the liver and suppresses VAT inflammation, suggesting their different effects on inflammation and metabolism [22]. In the literature by Hiromichi et al., the DPP-4 inhibitor vildagliptin reduced T2DM-induced increase in acute mortality after myocardial infarction with the restoration of autophagy [23], demonstrating the potential role of DPP-4 inhibitor in regulating autophagy. Nevertheless, the effect of DPP-4 inhibitor on hepatic steatosis remains variant. In a 24-week randomized controlled trial (RCT), sitagliptin was shown to not be more effective than placebo in improving hepatic steatosis and fibrosis in patients with NASH [24]. While Shahinul et al. reported that sitagliptin 100 mg once daily for 1 year ameliorates NAS by improving steatosis and ballooning, irrespective of diabetes [25].

Therefore, the present study was conducted to assess our hypothesis concerning the regulatory role of DPP-4 inhibitor in hepatic insulin resistance, inflammation and autophagy, and related metabolic dysfunction by daily gavage of sitagliptin in ob/ob mice, a well-established model of genetic T2DM and obesity. The results revealed that sitagliptin could attenuate obesity-induced hepatic insulin resistance and steatosis, suppress inflammation by regulating macrophage M1/M2 polarization imbalance, and promote liver autophagy via AMPK/mTOR signaling pathway.

## 2. Materials and Methods

### 2.1. Animal Experiments

Five-week-old male leptin-deficient homozygous ob/ob T2DM obese mice (B6.V-Lep^ob^/J) and their age-matched wild-type control (WT wild-type: C57BL/6J) were purchased from Beijing HFK Bioscience Co. Ltd. All mice were individually bred in-house on a 12 h light-dark cycle (light on at 7 am) and offered standard water and chow ad libitum at the Center for Laboratory Animal, Tongji Medical College, Huazhong University of Science and Technology. After a week for acclimatization at six-week-old, the ob/ob mice were randomly divided into the ob/ob group (saline, 50 mg/kg daily by gastrogavage) and the sitagliptin treatment group (ob/ob + Sita) (sitagliptin, 50 mg/kg daily, by gastrogavage), and the WT groups were given the equivalent dose of saline, 8 in each group, all were treated for 4 weeks [26].

All experiments were conducted in accordance with the Institutional Animal Care and Use Committee at the Tongji Medical College, Huazhong University of Science and Technology.

### 2.2. General Metabolic Phenotyping

Body weights were recorded daily. The level of serum insulin was detected by ELISA assays (AlpcoDiagnostics, Windham, NH). Serum and liver triglycerides and serum total cholesterol (TC) were measured using metabolic assay kits (Nanjing Jiancheng Bioengineering Institute, Jiangsu, China). The assays were carried out according to the manufacturer's specifications. Additionally, homeostasis model assessment index-insulin resistance (HOMA-IR) was calculated by using fasting glucose and insulin with the following equation [27]: HOMA-IR = fasting glucose (mmol/L) × fasting insulin (mU/L)/22.5.

### 2.3. Glucose and Insulin Tolerance Tests

At the end of week 2 and week 4, mice were fasted for overnight and intraperitoneally injected with 50% glucose (2.0 g/kg body weight) for the glucose tolerance test (GTT). Blood was collected from the tail to detect blood glucose before and at 30, 60, and 120 min after the injection. For the insulin tolerance test (ITT), mice were fasted for 6 hours and injected with insulin (1.0 U/kg body weight) (Novolin R; Novo Nordisk, Bagsværd, Denmark). Blood glucose levels were measured from the tail vein before and at 15, 30, 60, and 90 min after the injection. The area under the curve (AUC) was calculated as described previously [28].

### 2.4. Measurement of Inflammation

Plasma proinflammatory cytokines, including IL-6 and TNF-*α*, were quantified by the mouse ELISA kits (Eton Bioscience Inc., San Diego, CA) according to the manufacturer's instructions. To determine the NF-*κ*B P65 activity in the liver, the mouse NF-*κ*B P65 ELISA kit (Cayman Chemical, Michigan, USA) was, respectively, used according to the manufacturers' recommendations. The assay of activator protein 1 (AP1) activity was quantified by an ELISA-based assay according to a previous research [29]. All samples were run in duplicate and analyzed on the same day to minimize day-to-day variation.

### 2.5. Histological Evaluation

Sections (4 *μ*m thick) of paraformaldehyde-fixed, paraffin-embedded liver samples were stained with H&E for histological evaluation. A Motic microscope BA310 (Ted Pella Inc., Los Angeles, CA) equipped with a digital camera was applied to visualize and capture all the images. The degree of lipid droplets of the immunohistochemical images was then determined with Image-Pro Plus 6.0 software (Media Cybernetics Inc., CA, USA).

### 2.6. Total RNA Extraction and Real-Time Quantitative PCR (RT-qPCR)

Total RNA extraction from liver tissues was using Trizol reagent (Qiagen, Valencia, CA), and cDNA was synthesized from 2 *μ*g of total RNA using the PrimeScript RT reagent kit (Takara Biotechnology Co. Ltd., Japan). Then, real-time quantitative PCR (RT-qPCR) was performed with SYBR Green real-time PCR Master Mix (Takara Biotechnology Co. Ltd., Japan) at a LightCycler480-PCR machine (Roche Diagnostics, Mannheim, Germany). The comparative 2^−ΔΔCt^ method was applied to determine gene expressions relative to the standard housekeeping genes *Gapdh*, as described previously [30]. The primers for each gene, synthesized by Sangon Biotech (Shanghai, China), are presented in Table S1.

### 2.7. Western Blotting

Western blot analysis was performed as previously described [7]. Briefly, proteins were prepared from liver tissues using RIPA lysis buffer containing protease inhibitors and phosphatase inhibitors (Beyotime, Shanghai, China). Tissue protein concentrations were determined by bicinchoninic acid (BCA) kit (Beyotime, Shanghai, China). Homogenates were separated by SDS-PAGE and transferred to polyvinylidene difluoride membranes (Immobilon; Millipore, Bedford, MA, USA). The membranes were blocked in 5% bull serum albumin (BSA) solution or 5% nonfat milk at room temperature for 1 hour, then incubated overnight at 4°C with primary antibodies against GAPDH, AKT (insulin-stimulated), p-AKT (Ser473, insulin-stimulated), JNK, p-JNK (Ser63, Ser73), AMPK, p-AMPK (Thr172), mTOR, p-mTOR (Ser2448) (Cell Signaling Technology, Beverly, MA, USA), and LC3B (Sigma-Aldrich, USA). Secondary antibody was HRP-conjugated anti-rabbit or anti-mouse IgGs (Beyotime, Shanghai, China).

### 2.8. Statistical Analysis

In this study, all statistical analyses were performed using SPSS 22.0 (SPSS, IBM, Chicago, IL, USA) and GraphPad Prism 7 (La Jolla, CA, USA). All data were presented as mean ± standard error of the mean (SEM). Statistical significance between two groups was determined by the Student's *t*-test. Comparisons among several groups were performed by one-way ANOVA. *p* < 0.05 was considered statistically significant in all statistical tests.

## 3. Results

### 3.1. Sitagliptin Protects the ob/ob Mice from Body Weight Gain and Glucose-Lipid Metabolic Disorder

**Figure 1:**
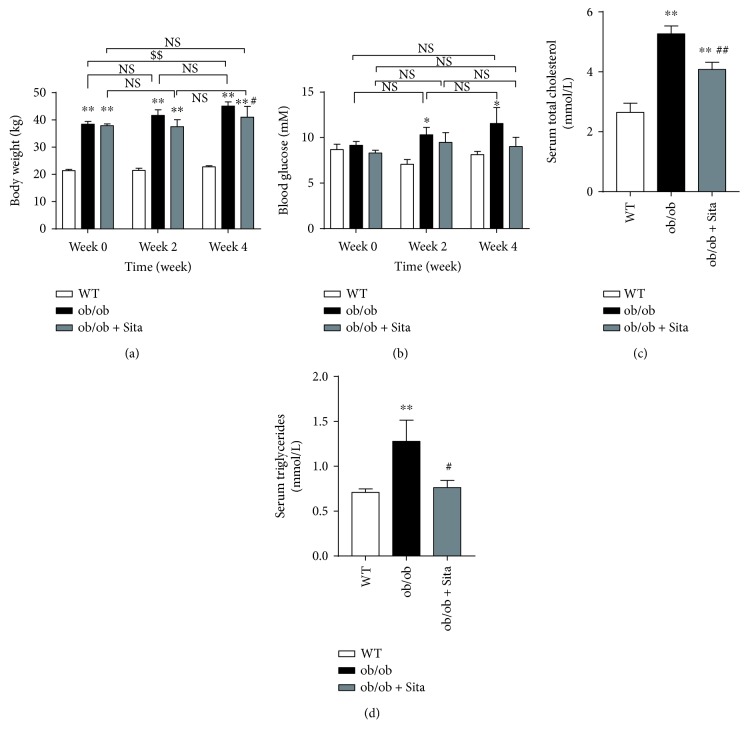
Sitagliptin protects the ob/ob mice from body weight gain and glucose-lipid metabolic disorder. Body weight (a) and fasting blood glucose (b) at different periods after sitagliptin treatment (*n* = 4–8). Serum total cholesterol (c) and triglycerides (d) at week 4 after sitagliptin treatment (*n* = 5-6). ^∗^ *p* < 0.05 and ^∗∗^ *p* < 0.01 versus WT group. ^#^ *p* < 0.05 and ^##^ *p* < 0.01 versus ob/ob group. ^$$^ *p* < 0.01 versus week 0. NS: not significant. All data are presented as mean ± SEM.

At the beginning of the treatment (week 0), ob/ob mice (ob/ob group and ob/ob + Sita group) showed significant higher body weight (39.14 ± 2.35 g) than that of the age-matched WT controls (21.39 ± 1.35 g, Figure 1(a)), but no significant differences in the blood glucose levels among the three groups (Figure 1(b)). As the experiment went on, ob/ob group showed a significant elevation of body weight (Figure 1(a)) and fasting blood glucose (Figure 1(b)) compared with WT group. Sitagliptin-treated group showed a slight but significant reduction in body weight and not significant decrease in fasting plasma glucose at week 4 compared with the ob/ob group. However, there was no significant change in their own body weight and fasting plasma glucose throughout the experiment (Table 1 and Figures 1(a) and 1(b)). To examine whether sitagliptin treatment affected lipid metabolism, we monitored parameters of serum total cholesterol (TC) and triglycerides (TG), these analyses revealed sitagliptin treatment showed a marked reduction of serum TC (Table 1 and Figure 1(c)) and TG (Table 1 and Figure 1(d)) compared with the ob/ob group.

### 3.2. Sitagliptin Ameliorates Systemic and Hepatic Insulin Resistance

**Figure 2:**
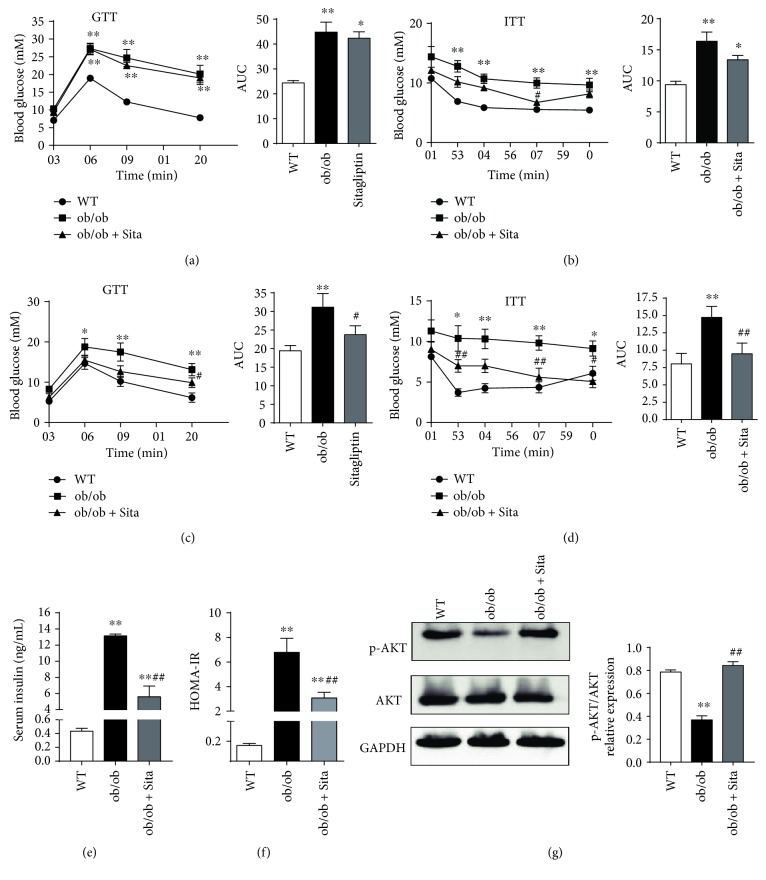
Sitagliptin ameliorates systemic and hepatic insulin resistance. Glucose tolerance test (GTT) and insulin tolerance test (ITT) were performed, respectively, at week 2 (a, b) and week 4 (c, d) (*n* = 4–8). Insulin sensitivity was analyzed by HOMA-IR (e) and fasting serum insulin (f) at week 4 (*n* = 4–6). Western blotting of insulin-stimulated AKT phosphorylation in the liver (g). The level of p-AKT was normalized to that of AKT (*n* = 8). ^∗^ *p* < 0.05 and ^∗∗^ *p* < 0.01 versus WT group. ^#^ *p* < 0.05 and ^##^ *p* < 0.01 versus ob/ob group. All data are presented as mean ± SEM.

We performed glucose tolerance test (GTT) and insulin tolerance test (ITT) on all mice at week 2 and the end of the treatment period (week 4). The area under the curve (AUC) was calculated to quantify glucose and insulin tolerance. GTT and ITT revealed that insulin resistance was markedly exacerbated in ob/ob mice (Figures 2(a) and 2(d)). The AUC of GTT showed a 6% decreased tendency and 23% decline after sitagliptin at week 2 (Figure 2(a)) and week 4 (Figure 2(c)), respectively, indicating that glucose intolerance was improved. ITT also showed that the ob/ob + Sita group had increased insulin sensitivity compared with the ob/ob group (Figures 2(b) and 2(d)). Moreover, the decrease of AUC_GTT_ and AUC_ITT_ was partial at week 2 and pronounced to be statistically significant at week 4 (Figures 2(a) and 2(d), *p* < 0.05). Furthermore, at the end of this experiment, the ob/ob group showed obviously increased HOMA-IR index compared to the WT group, which was markedly decreased after sitagliptin treatment (Figure 2(e), *p* < 0.01). Sitagliptin also suppressed fasting hyperinsulinemia (Figure 2(f), *p* < 0.01) as well as enhancing insulin signaling in the liver of ob/ob mice, associated with increased insulin-induced AKT phosphorylation (Figure 2(g), *p* < 0.01).

### 3.3. Sitagliptin Improves Hepatic Steatosis in ob/ob Mice

**Figure 3:**
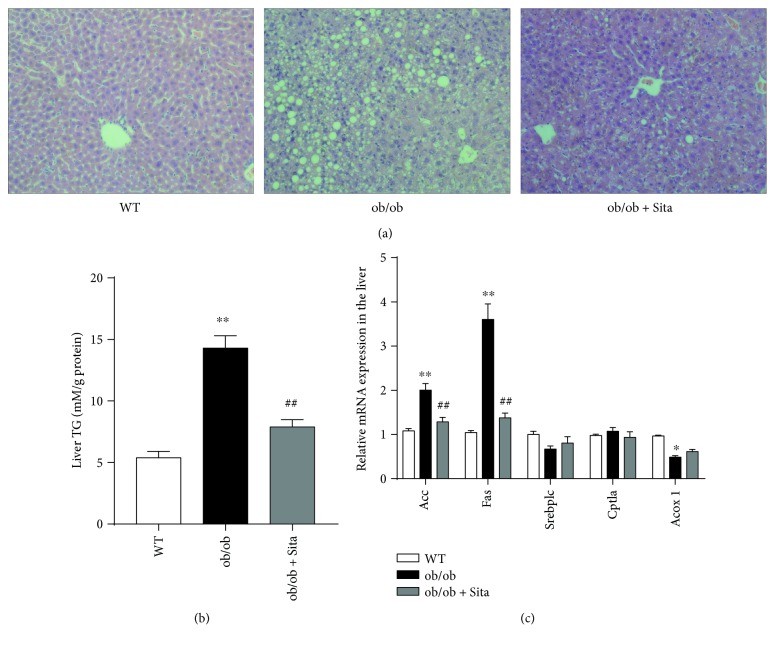
Sitagliptin improves hepatic steatosis in ob/ob mice. Lipid accumulation in the liver was evaluated by H&E staining (a). Original magnification ×200. Liver TG content (b) was measured (*n* = 8). Relative mRNA expressions of lipogenic regulator genes and fatty acid oxidation genes in the liver (c) were measured by RT-qPCR (*n* = 8). ^∗^ *p* < 0.05 and ^∗∗^ *p* < 0.01 versus WT group. ^##^ *p* < 0.01 versus ob/ob group. All data are presented as mean ± SEM.

We next examined whether sitagliptin treatment affected the development of hepatic steatosis. The histological analysis revealed that liver tissues of the ob/ob group showed more and larger size of lipid droplets on hematoxylin and eosin (H&E) staining sections, which were decreased markedly by sitagliptin (Figure 3(a)). These findings were associated with less liver triglyceride (TG) content in the ob/ob + Sita group (Table 1 and Figure 3(b), *p* < 0.01). In addition, we identified an obvious downregulation of hepatic lipogenic gene expression (*Acc* and *Fas*), while *Srebp1c* and genes involved in fatty acid oxidation (*Cpt1a* and *Acox1*) were unaltered (Figure 3(c)).

### 3.4. Sitagliptin Attenuates Systemic and Hepatic Proinflammatory Responses

**Figure 4:**
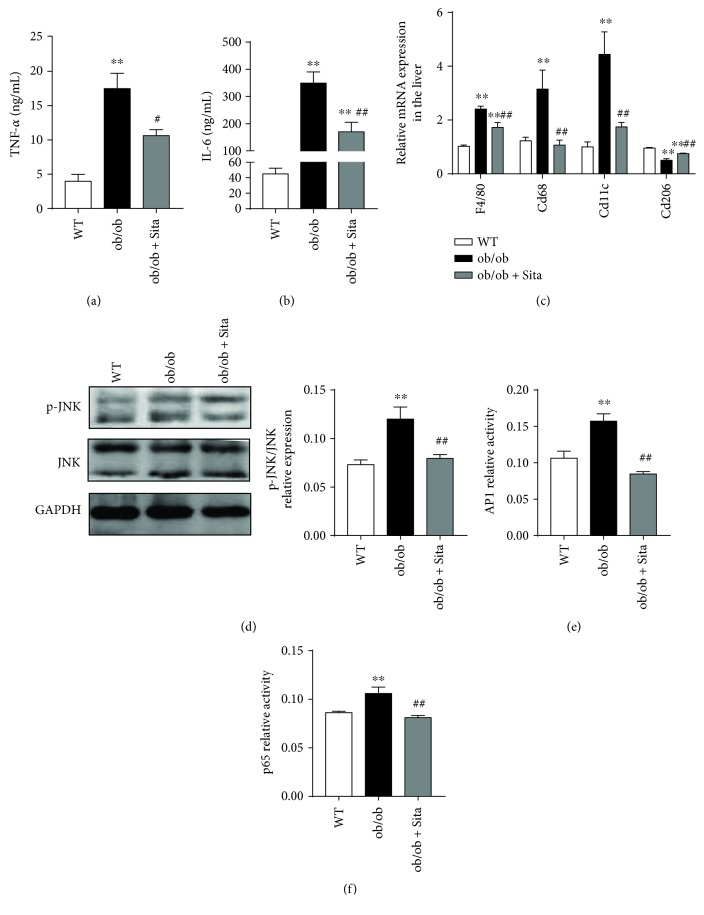
Sitagliptin attenuates systemic and liver inflammation in ob/ob mice. Serum inflammatory cytokines TNF-*α* (a) and IL-6 (b) were determined by ELISA (*n* = 3–6). RT-qPCR of markers (*Cd68*, *F4/80*, *Cd11c*, and *Cd206*) of total and M1/M2 macrophages (c) in the liver (*n* = 8). Gene expression was normalized to that of the *Gapdh* gene. Western blotting of phosphorylated JNK (p-JNK) and their total proteins (d) in the liver (*n* = 8). Representative Western blot images and graphs were representing the ratio of the phosphorylated protein of interest on total proteins. DNA binding activity of AP1 (e) and NF-*κ*B p65 activity (f) were determined from liver tissue extracts by ELISA for each sample relative to the normal control (*n* = 8). ^∗∗^ *p* < 0.01 versus WT group. ^#^ *p* < 0.05 and ^##^ *p* < 0.01 versus ob/ob group. All data are presented as mean ± SEM.

Next, we explored the effect of sitagliptin treatment on systemic and hepatic inflammation. As expected, the concentration of inflammatory cytokines TNF-*α* (Figure 4(a)) and IL-6 (Figure 4(b)) concentration were markedly lower in the ob/ob + Sita group compared with the ob/ob group (*p* < 0.05 and 0.01, resp.), indicating that the systemic inflammation was attenuated. In addition, sitagliptin significantly decreased the mRNA expressions of *Cd68*, *F4/80*, and M1 marker (*Cd11c*) and increased the M2 marker *Cd206*, showing the reduced number of infiltrated macrophages and attenuated M1/M2 polarization imbalance in the liver (Figure 4(c), *p* < 0.01). To elucidate the molecular mechanism, we detected the activation of NF-*κ*B and JNK/AP1 pathway. We found that the phosphorylation of JNK (Figure 4(d)) and AP1-binding activity (Figure 4(e)) was significantly reduced in ob/ob mice under sitagliptin treatment (*p* < 0.01), suggesting its role in preventing the activation of the inflammatory JNK/AP1 pathway. This effect was accompanied by a 23.5% decrease in the activity of NF-*κ*B p65 (Figure 4(f), *p* < 0.01).

### 3.5. Sitagliptin Upregulated Autophagy in the Liver

**Figure 5:**
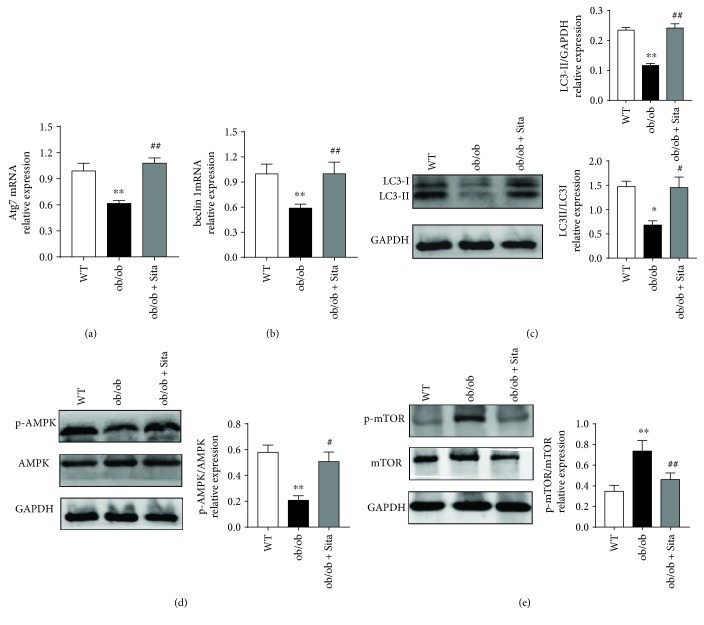
Sitagliptin enhanced autophagy in the liver. RT-PCR of autophagy components *Atg7* (a) and *Beclin1* (b) in the liver. Gene expression was normalized to that of the *Gapdh* gene (*n* = 8). Western blotting of LC3 (c), AMPK phosphorylation (d), and mTOR phosphorylation (e) in the liver. Representative Western blot images and graphs were representing the ratio of the phosphorylated protein of interest on total proteins or the ratio of LC3-II/GAPDH and LC3-II/LC3-I as measured by densitometry (*n* = 8). ^∗^ *p* < 0.05 and ^∗∗^ *p* < 0.01 versus WT group. ^#^ *p* < 0.05 and ^##^ *p* < 0.01 versus ob/ob group. All data are presented as mean ± SEM.

To further determine whether sitagliptin treatment modulate cell autophagy in the liver, we detected marker molecules of autophagic activities. The mRNA expressions of autophagic marker *Atg7* and *Beclin1* were reduced in the liver of ob/ob mice (*p* < 0.05 and 0.01, resp.). Sitagliptin treatment showed a proautophagic effect in the liver since it was able to upregulate mRNA expression of *Atg7* and *Beclin1* (Figures 5(a) and 5(b)). Conversion of the autophagy mediator LC3 from its long form to a shorter form (LC3-I) and subsequent fatty acid conjugation (LC3-II) is essential for its entry into the autophagosome membrane. Thus, the ratio of LC3-II to LC3-I is an indicator of autophagy induction [31]. As shown in Figure 5(c), LC3-II/GAPDH ratio was significantly lower in the ob/ob group when compared to the WT group (*p* < 0.01). Changes in LC3-II/LC3-I ratio were similar to those in LC3-II/GAPDH, though the difference was less statistically significant (*p* < 0.05). LC3-II level and LC3-II/LC3-I ratio were increased after sitagliptin treatment, suggesting that the reduction in autophagosome formation in ob/ob mice was significantly prevented by sitagliptin treatment. As is known, autophagy is stimulated by the energy sensor AMP-activated protein kinase (AMPK), and we found a significant reduction of phospho-AMPK and increase of phospho-mTOR in the ob/ob group (Figure 5(d), *p* < 0.01), while sitagliptin treatmen t significantly increased the phosphorylation of AMPK (Figure 5(d), *p* < 0.05). In addition, mammalian target of rapamycin (mTOR) is a negative regulator of autophagy, and phosphorylation of mTOR was elevated in the ob/ob group while decreased after sitagliptin treatment (Figure 5(e), *p* < 0.01).

## 4. Discussion

In the present study, we showed that in a genetic mouse model of obesity and insulin resistance, daily intragastric administration of sitagliptin for 4 weeks attenuates glucose-lipid metabolic dysfunction and hepatic steatosis. In addition, sitagliptin protects ob/ob mice against inflammation and insulin resistance, through reduction of macrophage accumulation, regulation M1/M2 macrophage polarization, and prevention of the inflammatory pathway in the liver, concurrent with improved autophagic processes.

Our study shows that 4-week treatment with sitagliptin is able to prevent the aggravation of weight gain and improve glucose tolerance and insulin sensitivity and lowered fasting serum insulin without reducing fasting plasma glucose in ob/ob mice (Figures 1 and 2). We also found that sitagliptin promotes a slight but no significant decrease of body weight, AUC_GTT_, and AUC_ITT_ at week 2. Thus, the length of the treatment may be critical in achieving better body weight and glucose control. Insulin signaling downstream AKT pathway in the liver has emerged as critical for the emergence of hepatic insulin resistance. In this investigation, we show that sitagliptin increases the insulin-stimulated phosphorylation of AKT in the liver (Figure 2(g)). This observation suggests that sitagliptin is able to improve hepatic insulin resistance in obese state.

Accumulating evidence demonstrates that the hepatic steatosis linked to insulin resistance is associated with elevation in lipid synthesis and altered hepatic fatty acid oxidation [32]. Our current data show that the development of hepatic steatosis was dramatically improved in ob/ob mice under sitagliptin administration (Figures 3(a) and 3(b)). In addition, hepatic mRNA expression of lipogenic markers *Acc* and *Fas* was downregulated with sitagliptin; however, *Srebp1c* and genes involved in fatty acid oxidation (*Cpt1a* and *Acox1*) were unchanged (Figure 3(c)). Our findings suggest that the hepatic protective effects of DPP-4 inhibition may be mediated through suppression of de novo lipogenesis rather than fatty acid oxidation. Liver macrophages are critical mediators of inflammation during the onset of NAFLD, and their depletion attenuates the progression of nonalcoholic steatohepatitis and insulin resistance [33]. At present, our data show that increased macrophage accumulation occurs in the liver of ob/ob mice with elevated *Cd68* and *F4/80* expression (Figure 4(c)). Besides, increased M1 marker *Cd11c* and decreased M2 marker *Cd206* expression indicated that macrophages in the liver shift towards a proinflammatory polarization in genetic obesity.

Sitagliptin reduced inflammatory cytokines and improved the proinflammatory M1 phenotypes of peripheral blood monocytes in T2D patients [34], as was shown in the current study that cytokines TNF-*α* and IL-6 were lower (Figures 4(a) and 4(b)), and proinflammatory M1 mRNA expression was downregulated while anti-inflammatory M2 upregulated in sitagliptin-treated ob/ob mice (Figure 4(c)). Sitagliptin treatment was shown to inhibit NF-*κ*B activation (Figure 4(f)), which are consistent with a previous study that anagliptin prevented LPS-induced elevations of inflammatory cytokines in macrophages, adipocytes, and the liver in mice, through suppression of NF-*κ*B transcriptional activity [35]. Related studies have shown that another vital intracellular proinflammatory pathway JNK/AP1 system is involved in insulin resistance and can mediate lipid-induced metabolic stress [36]. As shown in Figures 4(d) and 4(e), sitagliptin significantly suppressed both increases of phosphorylation of JNK and AP1 activities, which appear to be obviously enhanced in ob/ob mice, underlying the potential mechanism of sitagliptin-induced suppression of proinflammatory cytokines.

In addition, our data showed that the improved insulin sensitivity and inflammation in sitagliptin-treated ob/ob mice were in parallel with enhanced autophagy in the liver (Figures 5(a) and 5(c)). The AMP-activated protein kinase (AMPK) has been shown to play a vital role in protecting against obesity-induced insulin resistance and autophagy [37]. Our study shows that sitagliptin can activate AMPK in the liver tissue (Figure 5(a)). It is reported that the activation of AMPK promotes starvation-induced autophagy through inhibition of mTOR [38], as our data showed that sitagliptin prevented the increase in phosphorylation of mTOR in ob/ob mice (Figure 5(e)), enhancing hepatocellular autophagy. These findings reveal that sitagliptin promotes autophagy through activation of the AMPK/mTOR pathway. Moreover, hepatic lipid accumulation could be ameliorated via activating of autophagy [39]. Thus, the attenuation of hepatic steatosis in ob/ob mice with sitagliptin treatment (Figures 3(a) and 3(b)) may also be explained by the restoration of hepatic autophagy.

Serum levels of GLP-1 were not measured nor was the serum and hepatic DPP-4 activity, so inhibition of DPP-4 by sitagliptin was not assessed, which is one of the limitations of the present study. We also did not treat the animals with different doses of sitagliptin. Therefore, future studies should be conducted to confirm that DPP-4 inhibitor improves liver insulin resistance by suppressing inflammation and enhancing autophagy, focusing on the activity of GLP-1 and DPP-4 in both serum and tissues with different doses of sitagliptin treatment.

## 5. Conclusions

Our study provides evidence that sitagliptin can effectively ameliorate the progression of obesity-induced insulin resistance and hepatic steatosis by suppressing inflammation, via regulating macrophage M1/M2 polarization and NF-*κ*B and JNK/AP1 pathway, enhancing autophagy through the AMPK/mTOR signaling pathway in the liver. In conclusion, sitagliptin might be regarded as a promising therapy against the development of insulin resistance and NAFLD. The underlying mechanism of these effects needs to be further investigated.

## Figures and Tables

**Table 1:** Effects of sitagliptin on body weight, glucose, and lipid metabolism at week 4.

	Parameters		WT	ob/ob	ob/ob + Sita
Glucose metabolism	FBG (mmol/L)		8.13 ± 0.88	11.55 ± 3.49	9.03 ± 2.01
	Insulin (pmol/L)		0.43 ± 0.10	13.14 ± 0.45^∗∗^	5.59 ± 2.65^##^
Lipid metabolism	Blood lipids (mmol/L)	TC	2.64 ± 0.76	5.27 ± 0.58^∗∗^	4.10 ± 0.59^##^
		TG	0.71 ± 0.10	1.28 ± 0.53^∗∗^	0.76 ± 0.20^#^
	Liver lipids (mM/g protein)	Liver TG	5.48 ± 1.76	13.70 ± 3.02^∗∗^	8.01 ± 1.98^##^

FBG: fasting blood glucose; TG: triglycerides; TC: total cholesterol. ^∗∗^ *p* < 0.01 versus WT group. ^#^ *p* < 0.05 and ^##^ *p* < 0.01 versus ob/ob group. *n* = 4–8 mice per group. Data are expressed as mean ± SEM.

## Abbreviations

T2D: Type 2 diabetes
TNF-*α*: Tumor necrosis factor-*α*
IL-6: Interleukin-6
JNK: Jun N-terminal kinase
IKK*β*: I*κ*B kinase
HFD: High-fat diet
DPP-4: Dipeptidyl-peptidase 4
GLP-1: Glucagon-like peptide 1
GIP: Glucose-dependent insulinotropic peptide
WAT: White adipose tissue
DIO: Diet-induced obese
RCT: Randomized controlled trial
WT: Wild-type
TC: Total cholesterol
TG: Triglycerides
GTT: Glucose tolerance test
ITT: Insulin tolerance test
AUC: Area under the curve
H&E: Hematoxylin and eosin
AP1: Activator protein 1
AMPK: AMP-activated protein kinase
mTOR: Mammalian target of rapamycin
NAFLD: Nonalcoholic fatty liver disease
HOMA-IR: Homeostasis model assessment index-insulin resistance
SEM: Standard error of the mean.

## Supplementary Materials

Supplementary Materials Table S1: primer sequences for qPCR analysis.
