# Supplementary material for: Dipeptidyl-Peptidase 4 Inhibitor Sitagliptin Ameliorates Hepatic Insulin Resistance by Modulating Inflammation and Autophagy in ob/ob Mice
Source: Int J Endocrinol. 2018 Jul 19;2018:8309723. doi: 10.1155/2018/8309723 (PMC6079465; doi:10.1155/2018/8309723)
Supplement: Supplementary Materials — Table S1: primer sequences for qPCR analysis. [file 8309723.f1.pdf]

Supplementary Materials

Table S1. Primer sequences for qPCR analysis

Target	Direction	Sequence
Gapdh	Forward	5'GGTGAAGGTCGGTGTGAACG 3'
	Reverse	5'CTCGCTCCTGGAAGATGGTG 3'
Acc	Forward	5'TTTCTTCCTTCGCCTCCTTT 3'
	Reverse	5'GCCAATCTCATTTCCTCCT 3'
Fas	Forward	5'AAATTCAGCCCGTTGGAGT 3'
	Reverse	5'AAGTTGCATCCACCCAAATC 3'
Srebp1c	Forward	5'GAGGCAGAGAGCAGAGATGG 3'
	Reverse	5'GACAAAGAGAAGAGCCAAGCA 3'
Cpt1a	Forward	5'TCAAGCCAGACGAAGAACATC 3'
	Reverse	5'TGGTAGGAGAGCAGCACCTT 3'
Acox1	Forward	5'ATCAAGAGAAGCGAGCCAGA 3'
	Reverse	5'CCGAGAAAGTGGAAGGCATA 3'
Cd68	Forward	5'CTTCGGGCCATGTTTCTCTT 3'
	Reverse	5'ATTGTCGTCTGCGGGTGAT 3'
F4/80	Forward	5'GCTGTGAGATTGTGGAAGCA 3'
	Reverse	5'GGCAAGACATACCAGGGAGA 3'
Cd11c	Forward	5'GGTGAAGGTCGGTGTGAACG 3'
	Reverse	5'CATCAGGGAGAACCGTGTG 3'
Cd206	Forward	5'TTCAGCTATTGGACGCGAGG 3'
	Reverse	5'GAATCTGACACCCAGCGGAA 3'
Atg7	Forward	5'ATGCCAGGACACCCTGTGAACTTC 3'
	Reverse	5'ACATCATTGCAGAAGTAGCAGCCA 3'
Beclin1	Forward	5'AGCCTCTGAAACTGGACACG 3'
	Reverse	5'TAGCCTCTTCCTCCTGGGTCT 3'
